# Improving the Event-Based Classification Accuracy in Pit-Drilling Operations: An Application by Neural Networks and Median Filtering of the Acceleration Input Signal Data

**DOI:** 10.3390/s21186288

**Published:** 2021-09-19

**Authors:** Sarahi Nicole Castro Pérez, Stelian Alexandru Borz

**Affiliations:** Department of Forest Engineering, Forest Management Planning and Terrestrial Measurements, Faculty of Silviculture and Forest Engineering, Transilvania University of Brasov, Şirul Beethoven 1, 500123 Brasov, Romania; sarahi.castro@unitbv.ro

**Keywords:** classification accuracy, improvement, acceleration signal, median filtering, artificial neural networks, regularization parameter, tunning, forestry, planting, pit-drilling operations

## Abstract

Forestry is a complex economic sector which is relying on resource and process monitoring data. Most of the forest operations such as planting and harvesting are supported by the use of tools and machines, and their monitoring has been traditionally done by the use of pen-and-paper time studies. Nevertheless, modern data collection and analysis methods involving different kinds of platforms and machine learning techniques have been studied lately with the aim of easing the data management process. By their outcomes, improvements are still needed to reach a close to 100% activity recognition, which may depend on several factors such as the type of monitored process and the characteristics of the signals used as inputs. In this paper, we test, thought a case study on mechanized pit-drilling operations, the potential of digital signal processing techniques combined with Artificial Neural Networks (ANNs) in improving the event-based classification accuracy in the time domain. Signal processing was implemented by the means of median filtering of triaxial accelerometer data (window sizes of 3, 5, and up to 21 observations collected at 1 Hz) while the ANNs were subjected to the regularization hyperparameter’s tunning. An acceleration signal processed by a median filter with a window size of 3 observations and fed into an ANN set to learn and generalize by a regularization parameter of α = 0.01 has been found to be the best strategy in improving the event-based classification accuracy (improvements of 1% to 8% in classification accuracy depending on the type of event in question). Improvement of classification accuracy by signal filtering and ANN tuning may depend largely on the type of monitored process and its outcomes in terms of event duration; therefore, other monitoring applications may need particular designs of signal processing and ANN tuning.

## 1. Introduction

Given its complexity generated by the resources, business, management types and operational diversity, forestry is one of the economic sectors that could benefit from the use of the latest technology to enhance its overall effectiveness. There are some examples documented in the literature on the use of electronics [1], sensor-based and machine learning techniques in forestry, which have set the stage for the implementation of big data analytics and artificial intelligence in forestry [2]. In addition, there have been implemented many innovation projects, and many initiatives were taken with the aim of exploiting the latest developments in technology for forestry-related purposes [3,4,5]. However, the situation cannot be characterized as being a generalized one since the development and effective implementation of these kinds of technologies are generally running slower in forestry, leaving many of the countries at their infantile stage in relation to their use.

A typical example is that of Romania, which exhibits a complex business model in forestry that interconnects many processes and stakeholders, at least in the wood supply chain [6]. In the country, forests are currently managed for their multiple values and services provided, including wood production, while the forestry- and forest-related economies significantly contribute to the country’s GDP [7]. Most of the resource and process monitoring activities could benefit to a large extent from using the latest technologies of the artificial intelligence and machine learning to support real-time decision making and to set the ground for improvements. While no studies were identified to evaluate the industry’s needs for such technologies in Romania, some small-scale tests have already proven their usefulness, in terms of cost saving and safety [8,9,10]. In addition, the operational level has been identified in international forestry to be one of the potential beneficiaries of sensor-based and machine learning implementations [8,9,10,11,12], which enabled significant resource savings and safety improvements. At this level, manual-dominated tasks have been approached in forestry under the umbrella of the so-called human activity recognition, which has been implemented by the use of various data collection platforms and machine learning techniques e.g., [11,12]. A similar approach has been used to monitor tool or machine-supported tasks, at least when such machines were not equipped with built-in production monitoring systems [8,9,10,13]; this approach still justifies its relevance due to the low to intermediary mechanization level of forest operations that still prevails in many parts of the world [14,15,16].

As a baseline, modern operational monitoring techniques were aimed at documenting relevant events in the time domain and their results were often checked against the outcomes of traditional time studies so as to be able to evaluate their effectiveness. Both approaches are using some sort of time classification, which turns out to be important for comparison and modeling studies supporting various relevant goals in forestry [17]. To this end, the effectiveness and usefulness of automated time studies were described to be strongly related to their used procedures, methods and tools’ availability, generalization ability, maintainability and reliability [18]. However, a full comparability between the manual and automated techniques is often difficult to make, given both the limited human capability and the limitations of automated procedures. Therefore, from the perspective of a machine-learning application in operational monitoring, one of the most important problems to be solved is that related to the events’ classification performance in the time domain, with the aim of reaching close to a full classification accuracy. Artificial intelligence (AI) and machine learning (ML) techniques are gaining popularity as a result of their efficacy, precision and speed. One of the interesting features of the ML techniques is their excellent ability in working with large data sets in less time and with more accuracy than traditional methods. An essential aspect of AI has been its ability to forecast, which helps to reduce expenses and, hence, to enhance revenue [19]. The growth of the service sectors, as well as the growth of stored and live data (big data), requires the use of artificial intelligence. The fact that a human cannot perform the tasks of a machine in a natural way makes AI a complement to global development. It is likely, therefore, that artificial intelligence will soon be present in every aspect of our life since it can be already found in a wide range of industries, including medicine [20,21,22,23], communication [24,25,26,27], marketing [28,29,30], agriculture [31,32,33] and, of course, forestry [8,9,10,11,12,13,34,35]. In relation to operational monitoring by the means of automated time studies, recent research on the topic in forestry have proven that high classification accuracies may be achieved by the use as inputs in the machine-learning algorithms of raw signals outputted by various type of sensors such as accelerometers, gyroscopes and sound-pressure level meters [8,9,10,11,12,13]. In addition, signals outputted by accelerometers coupled with ML techniques have proven very useful not only in the forestry but also in other engineering disciplines such as those dealing with infrastructure and its monitoring [36,37,38]. While the approach of using the raw data as inputs may prove to be useful for fully automated, real-time applications, because it may ease the computational effort, in many ways its use in a processed form for offline modeling and improvement is important for the science. In addition, there are hints that processing of the raw signals and parameter tunning in machine learning applications may be useful to improve the outcomes in terms of classification accuracy; in particular, the use of median filters, tuning the model size and its learning rates could be among the good strategies to increase the classification performance [8,9,10,11,13].

The aim of this study was to check if the classification accuracy of events recorded in the time domain as specific to mechanized pit-drilling operations could be enhanced by digital signal processing techniques and hyperparameter tunning of one class of machine learning techniques—Artificial Neural Networks (ANNs). To this end, the paper was designed as a case study on triaxial accelerometer data collected in mechanized pit-drilling operations. The data were processed by regular median filters and fed into ANN algorithms by a trial-and-error approach so as to tune the learning regularization parameter and to keep the learning and generalization errors at minimum.

## 2. Materials and Methods

### 2.1. Description of the Datasets and of the Underlying Operations

**Figure 1:**
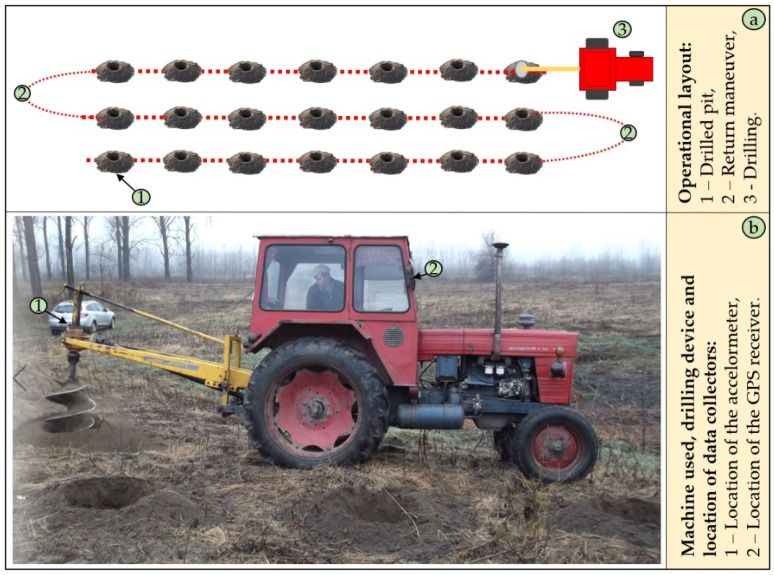
Operational layout and the equipment used. Legend: (**a**) the general operational layout; (**b**) pit-drilling equipment and location of the dataloggers during the field data collection.

The data supporting this study was collected in the southwestern part of Romania (Dolj county) covering 4 operational days (21, 22, 24 and 27 November 2018). The data was collected and documented by the use of a video camera and of an Extech VB 300 triaxial accelerometer (Extech Instruments, FLIR Commercial Systems Inc., Nashua, NH, USA). To document the locations of the study, a GPS unit (GPSmap 62stc, Garmin International Inc., Olathe, KS, USA) was placed outside the machine’s cab. The video camera was installed within the cab at a location where the driller’s activity could be readily seen, and it was set to continuously record the operations. The acceleration datalogger was mounted on the driller’s transmission to measure the acceleration based on the vibration emitted during the operations, and it was set to collect data at a sampling rate of 1 second. A description of the general operational layout, drilling and data collecting equipment is given in Figure 1.

The total number of observations recorded by the triaxial accelerometer was close to 100,000; however, after artifacts’ removal and signal processing steps, a number of 83,685 observations was kept for analysis (see Section 2.2). In the area of study, poplar planting operations were done in two successive steps, namely a mechanized pit-drilling by the use of a Selvatici (Selvatici, San Lazzaro di Savena, Italy) driller powered by a UTB 650 (UTB, Braşov, Romania) tractor (Figure 1), followed by a manual seedling planting at a scheme of 3 × 2 m. The pits were done at the dimensions enabled by the driller, and they were of ca. 60 cm in depth and ca. 60 cm in diameter. Based on the tasks documented by video recording, in the office phase of the study were identified events such as drilling the pits, machine stopped with the engine off, machine stopped with the engine on and machine moving, which characterized the mechanized part of the poplar planting operations. Most of these events (i.e., drilling and moving) occurred on a cyclic basis, although there were some movements at the end of the plots and between the plots, which occurred less frequently. For consistency, three types of events were kept for further analysis and generically named as “Drilling”, “Other” and “Stopped”, because these could be largely classified as the main, complementary and delay times according to the time classification in forest operations [39].

### 2.2. Data Processing

Data processing consisted of several steps. A first step was that of labeling the data according to the three classes of operational events (“Drilling”, “Stopped” and “Other”), which was supported by the video files and the signal magnitudes stored in the acceleration dataset. For this purpose, the Euclidian Norm (EN) outputs of the accelerometer data were used for guidance. A second step was that of removing the artifacts such as the periods of time in which the dataloggers were placed on and taken down from the machine, machine driving from the nearby communities to the places of operation, and the time spent to go from one operated plot to another. By doing so, the dataset was brought closer to the operational reality, which supposes only the events that may occur in the operated plots or nearby, and it was termed as the EN (raw) dataset. A third step was that of applying signal processing techniques to the raw signal data by the means of regular median filtering. The sizes used for the median filters were odd numbers from 3 to 21 (M3 to M21), standing for the number of observations taken as a reference by the sliding window of each filter. Following this step, the data was retained as a final dataset, being further divided in two parts, namely a training dataset (TRAIN), which accounted for 80% of the data, and a testing dataset (TEST), which accounted for the rest of the data (20%). Based on the EN and median filtered data (M3 to M21), two classes of files were created, one standing for the training data and which contained 11 files (EN_TRAIN and M3_TRAIN to M21_TRAIN) and one standing for the testing data, also containing 11 files (EN_TEST and M3_TEST to M21_TEST).

As a general behavior, the raw signals were altered by filtering in the sense of removing the impulse noise to a degree, which depended on the median filter used. As a fact, this is one of the important properties of the median filters, along with their ability to preserve the edges of the data and avoid truncation [40,41]. One of median filter drawbacks is that some datapoints will be lost; data loss increased by the filter size, from two observations for M3 to 20 for the M21. Datasets of 83,685 observations were those obtained following the median filtering. All of the processing steps as described above were carried out in a Microsoft Excel^®^ workbook (Microsoft, Redmond, WA, USA, 2013 version).

### 2.3. Development of the Artificial Neural Networks

#### 2.3.1. Software Used to Develop the ANN Models

To develop the ANNs, Orange Visual Programming Software (version 3.2.4.1) was used, which is a user-friendly software application that can be downloaded and used for free [42]. The application integrates a set of widgets, allowing it to analyze large quantities of data using visualizable computational processes. In order to run any type of analysis, it is necessary to create a visual map (workflow), which is composed of various interconnected tools (widgets). “File”, “Neural Network” and “Test and Score” widgets are typically included in the training workflow of the ANN models. The files EN_TRAIN and M3_TRAIN to M21_TRAIN were fed successively as inputs in the “File” widget. The Neural Network widget enables the use of a multilayer perceptron (MLP) algorithm with backpropagation. Once connected to the previous widgets and executed, the “Test and Score” widget displays the metrics needed in the evaluation of the model’s performance. After running and scoring tasks, the model can be saved, an approach that has been taken to store the models into a computer. Each of the saved model was uploaded in the testing phase using the “Load Model” widget, which was connected to its corresponding testing file using a “File” widget. Then both widgets were linked to the “Predictions” widget, which was used to display a given model’s predictions on the data. These predictions were attached to the “Data table” widget in order to export this data into a Microsoft Excel^®^ format.

Apart of the Orange Visual Programming Software (version 3.2.4.1) used to run the ANNs, Microsoft Excel^®^ (Microsoft, Redmond, WA, USA, 2013 version) was used to tabulate the data, to run the median filtering procedures and to plot the results as graphs. The computer used in the study was a home computer with a basic performance (Dell Inspiron 15 7000, Dell Technologies, Austin, TX, USA), equipped with a Windows 10 Home operating system, an Intel^®^ Core™ i7-8550U CPU 1.80 GHz 1.99 GHz processor, 8.00 GB of RAM memory and a NVIDIA G-Force Graphic card.

#### 2.3.2. Architecture of the Artificial Neural Network

As the work of [43] indicated, it is recommended to configure the ANN’s size with high values of the depth and width. This approach was used to keep the ANN at its maximum size as enabled by the used software before the training and testing phases. As such, the general architecture of the ANN consisted of three hidden layers (depth) of 100 neurons each (width); the ANN models have used as inputs the datasets described in Section 2.2, in the form of a time-ordered sequence of either raw or median filtered observations. The output layer had three possible outcomes as described in Section 2.1, namely “Stopped”, “Drilling” and “Other”. Each of the 11 datasets was used for training by running 1,000,000 iterations. ReLu (the rectified linear unit function) was used as an activation function, based on its high performance in solving nonlinear complex problems [44,45]. The selected solver for weight optimization was Adam (the stochastic gradient-based optimizer), which was chosen based on its small training costs [46]. The only tuned hyperparameter was the regularization term (α, L2 penalty), which was set successively at 0.0001, 0.001, 0.01, 0.1, 1 and 10. Accordingly, the number of developed and saved ANN models for the training phase was of 66. In addition, a cross-validation was used for the training and scoring, supposing a stratified method with a number of 20 folds. The average training time was of ca. 19 min.

#### 2.3.3. Classification Performance Metrics

The performance metrics obtained from the training phase were grouped into a single Excel sheet. The data was saved as aggregated metrics (average over the classes) and also by classes (“Drilling”, “Other”, “Stopped”). Among the computed metrics of classification performance were the area under the curve (AUC), classification accuracy (CA), F1 score (F1), Recall (REC), Log loss, Specificity and the time needed to train the ANN. All of these metrics were saved for each of the trained ANN model. Full definitions, interpretation and the computational procedures of the metrics used can be found, for instance, in [47,48].

Once the models were trained and saved, the workflow continued with the testing phase, which was run on the data kept for this task (ca. 20% of the data, 11 files); each of the models developed for the values set for the α parameter (six models for EN_TRAIN and for M3_TRAIN to M21_TRAIN, respectively) was tested on its corresponding test dataset (EN_TEST and M3_TEST to M21_TEST). The same classification performance metrics and errors were computed in the testing phase, where the Log loss was used as a metric to evaluate the generalization errors. Following the testing phase, the probabilities of given data points to fall within a given class (“Drilling”, “Other”, “Stopped”) were extracted and plotted against the magnitude data of those datasets, following the procedure described in [13]. The criteria used for evaluating the training and testing models and for selecting the best alternative were the classification accuracy (CA) and the Log loss error. Nevertheless, the data was plotted for all the developed and tested models in a comparative approach so as to identify the eventual improvements brought by median filtering and regularization term tunning. An additional step was that of extracting the relevant correctly and misclassified data from the raw (EN), the best and the worst performing testing datasets so as to be able to compare their performance in this respect and to plot the probabilities outputted by the three ANN models for the testing data. These steps were done in the Orange software by the help of a confusion matrix, which was used to output the data in a data table widget and then to export it for processing and analysis in Microsoft Excel^®^. The way in which a confusion matrix can be constructed as well as the type of data included into it are explained, for instance, in [48].

## 3. Results

### 3.1. Description of the Labeled Dataset

The labeled data coming from the four days of field observations is summarized in Table 1 in the form of the number of observations taken into analysis per day and their absolute and relative frequencies in the labeled dataset, as true classes.

There were no large differences in the daily sample sizes used in analysis, but there were differences in terms of frequencies over the true classes. Accordingly, the first two days were characterized by highly unbalanced frequencies over the true classes, while the last two days have shown a relative balance in relation to the frequencies. According to Table 1, “Drilling” class of events (observations) accounted for almost 43% of the used dataset, while “Other” and “Stopped” events accounted for ca. 33 and 24%, respectively. Together, the data shown in Table 1 characterizes a class imbalance, which is typical in the time domain to pit-drilling as well as to other kind of operations.

### 3.2. Classification Performance and Errors during the Training Phase

**Figure 2:**
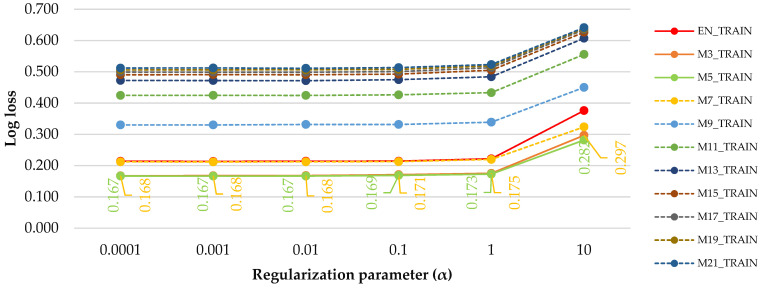
Log loss errors of the training datasets as a function of regularization parameter term and filter size. Legend: orange and green labels stand for the values of Log loss for M3_TRAIN and M5_TRAIN, respectively.

Figure 2 shows the effect of the regularization term’s tunning (α = 0.0001, α = 0.001, α = 0.01, α = 0.1, α = 1, α = 10) on the Log loss error accounting for the raw (EN_TRAIN) and median filtered data (M3_TRAIN to M21_TRAIN). Log loss was used to check which of the regularization parameter (α) and of the filtered dataset have led to the best performance in terms of training errors.

Three clusters may be identified in Figure 2 in relation to the values of Log loss errors and filter size. A first cluster was that of EN_TRAIN, M3_TRAIN, M5_TRAIN and M7_TRAIN, which were the datasets that performed the best in terms of errors. In this cluster, the best performing filtered datasets seemed to be the M3_TRAIN and M5_TRAIN, for which the Log loss errors were the lowest. The second cluster was that of M7_TRAIN, which stood apart from the rest of the datasets, and the third cluster was that of M11_TRAIN to M21_TRAIN, which were the datasets that performed the worst. 

For values of α of 0.0001 to 0.01, the errors of the M3_TRAIN and M5_TRAIN were kept at the same level, showing small differences in performance between the two filtered datasets. Similar, even though increased values, were preserved for α = 0.1. However, from α = 1, the Log loss error started to increase in relation to α. By considering the computational effort, the comparison gave the lowest errors and efforts for α = 0.01, which were of up to 0.167 (17%). Accordingly, by median filtering of the data, the errors can be decreased. However, this did not work for any filter size in the training phase. Only the filters of three to seven observations were among those outputting the highest performance, and for similar error rates, the M3 filter had the advance of preserving the lowest data loss in relation to the raw data and of easing the computational effort.

**Figure 3:**
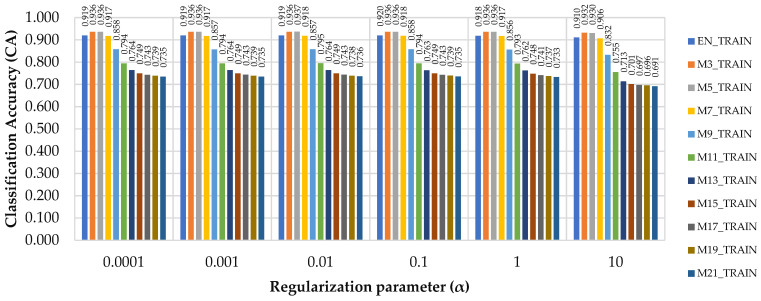
Classification Accuracy (CA) of the raw (EN_TRAIN) and filtered datasets (M3_TRAIN to M21_TRAIN) as function of regularization parameter (α).

Figure 3 shows the classification accuracy (CA) by considering the values given to the regularization parameter and the filtered datasets. A general trend can be observed in which the CA decreased as the value of α and of the window size increased. In all the cases which preserved the same value of α, the best performance was achieved for M3_TRAIN and M5_TRAIN, with only minor differences between the two for α = 0.01 and α = 10. These results are consistent with those given for the Log loss errors, showing that, besides the value set for the regularization term, the window used for data filtering is important for increasing the classification performance. Therefore, in the training phase, filtering data by M3 and M5 filters improved the classification accuracy by 1 to 2% (Figure 3), which was true for α values from 0.0001 to 1. For an α = 10, the improvement was even higher if one compares the values of CA for M3 and M5 against EN, but the value set for the regularization term has led, in general, to lower classification accuracies (Figure 3).

**Figure 4:**
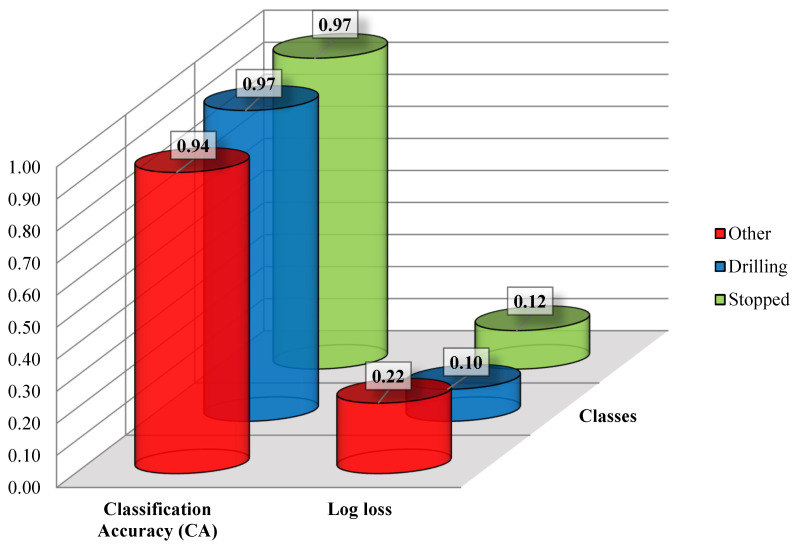
Representation of the classification accuracy (CA) and Log loss for the M3_TRAIN dataset by accounting for the three classes of events.

Figure 4 shows a comparison in terms of classification accuracies and Log loss errors in the training phase by using the M3_TRAIN data and α set at 0.01. The choice of this scenario for comparison was based on the general results shown in Figure 2 and Figure 3. As shown, the highest classification accuracies were those characterizing “Drilling” and “Stopped” classes, which accounted for 97%. The same classes have outputted the lowest training error (0.10 and 0.12, respectively). The “Other” class, on the other hand, had a classification accuracy of 94% and a Log loss error of 0.22. This result could be due to the fact that this class contained events such as the machine being stopped with the engine working and moving, respectively; therefore, the data might have been characterized by transient parts between these states. Accordingly, the main events such as the effective drilling and “Stopped” were characterized by even a higher classification accuracy and lower errors in the training phase.

### 3.3. Classification Performance and Errors during the Testing Phase

**Figure 5:**
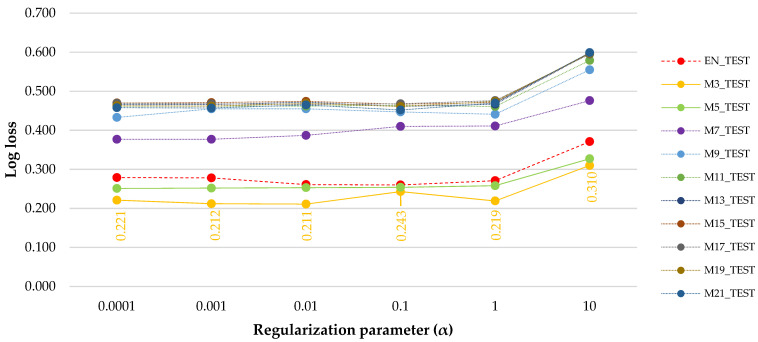
Log loss errors of the testing datasets as a function of regularization parameter term and filter size. Legend: the orange labels stand for the Log loss values of the M3_TEST dataset.

The results presented in Figure 5 are based on the predictions made by the saved ANN models over the testing data, which were different compared to the training phase. First of all, only two evident clusters were identified in relation to the Log loss errors plotted against the values of the regularization term. A first cluster contained the error values of EN_TEST, M3_TEST and M5_TEST, while the second one contained the rest of the datasets.

The lowest Log loss (generalization) error (0.211) was specific to a regularization parameter set at α = 0.01 when using the M3_TRAIN ANN model over its corresponding testing data, meaning that the M3_TEST had the lowest error. For the M5_TEST data, the Log loss errors were kept at approximately the same value for α values in the range of 0.0001 to 1, which was different if compared to the M3_TEST. The closest values of the Log loss errors between EN, M3 and M5_TRAIN were found for α = 0.1, probably indicating that the filtering by windows of three and five observations had little effect on the error in this case; however, they have exceeded the minimum error value of M3_TRAIN for α = 0.01.

**Figure 6:**
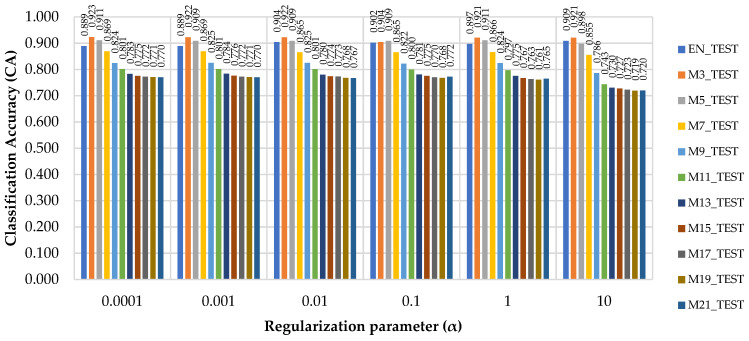
Classification Accuracy (CA) as a function of the regularization parameter (α) and median filters used.

Figure 6 compares the values of CA obtained in the testing phase of the raw (EN_TEST) and median filtered (M3_TEST to M21_TEST) data. As shown, in most of the cases the highest classification accuracy (CA) was found for the M3_TEST dataset. The best outcome in terms of classification accuracy (CA) was specific to α = 0.0001, where classification accuracy has reached 93%; however, the generalization errors for this value of the regularization parameter were higher as compared to α = 0.01. Testing phase has revealed a similar data pattern in terms of classification accuracy as a function of the median filter used and of the value set for the regularization parameter. Data consistency was also kept in the testing phase showing that for the type of operations taken into study the M3 filter performed the best in terms of classification accuracy and generalization error. The same rate of improvement (ca. 2%) in classification accuracy was found for α = 0.01 when comparing M3_TEST against EN_TEST.

**Figure 7:**
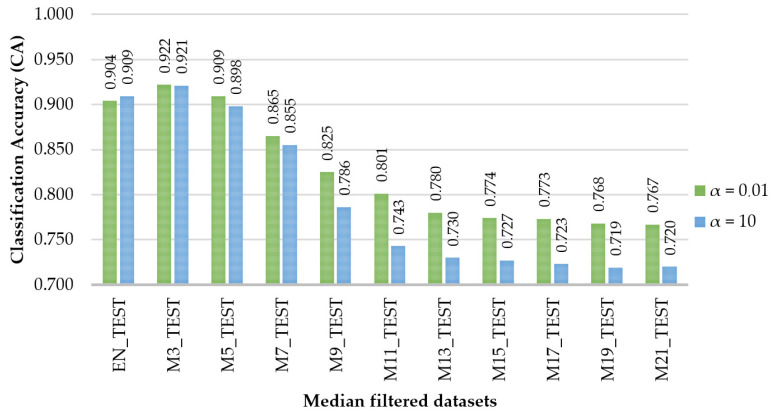
Variation in the classification accuracy (CA) of the test datasets as a function of the median filter size for the best and worst scenarios in terms of regularization parameter used.

However, the classification accuracies were lower, and the generalization errors were higher in the testing phase. The best (α = 0.01) and the worst (α = 10) scenarios in terms of generalization error (Log loss) are compared by their classification accuracies in Figure 7, by accounting for the median filter used. The figure enables a comparison of the effects that the value of the regularization term might have over the classification accuracy, showing that, for M3 and M5, they were rather similar, while for M7 to M21, increasing the value of α produced worse results for the same dataset in the testing phase.

### 3.4. Misclassifications and Probabilities

#### 3.4.1. Misclassification

During the testing phase, misclassified data is important to evaluate the model’s performance. In this study, such data was extracted for the testing phase for the EN_TEST, M3_ TEST and M21_ TEST by considering a regularization term of α = 0.01. The choice of these datasets was guided by the results found in the testing phase (Figure 5, Figure 6 and Figure 7), showing that α = 0.01 has provided the best generalization solution. The results from the confusion matrix were used to tabulate the data shown in Table 2.

The analysis of data given in Table 2 leads to the conclusion that there was a high difference in terms of correctly classified data, particularly for the data belonging to the “Other” event class. This class was found to hold the highest number of misclassifications, a finding that may be explained as in the case of the Log loss and classification accuracy (CA) results presented above. As such, different events contained in this class had a very wide variation in terms of acceleration magnitude, making the separation of this event difficult for the ANN models. According to the M3_TEST data, “Drilling“ and “Stopped“ had the lowest misclassification rates, which were also improved if compared to the EN_TEST and which could be interpreted as a contribution to the classification performance by the use of the three-observation filter. The same held true for the “Other” class, a case in which the misclassification decreased compared the EN_TEST. The highest gain in classification accuracy was that of the “Stopped” class, which, by filtering (M3) moved from 88% (EN_TEST) to 92% (M3_TEST). The M21_TEST data appeared to perform very good for the “Drilling” and “Stopped” classes, with the last one outputting the maximum classification accuracy, which was the effect of the window size of the filter. However, the high classification performance for the “Stopped” class was on the expense of the classification performance of the “Other” class.

#### 3.4.2. Probability Plots

**Figure 8:**
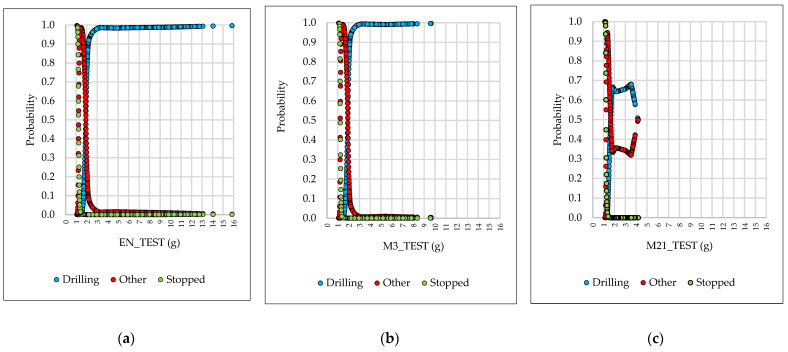
Selected plots showing the predicted classification probabilities. Note: (**a**) predicted classification probability of the EN_TEST dataset; (**b**) predicted classification probability of the M3_TEST dataset; (**c**) predicted classification probability of the M21_TEST dataset.

The classification probabilities were plotted for α = 0.01 because, in the training and testing phases, this was the regularization term characterizing the lowest Log loss errors and some of the highest classification accuracies (CA). For a case-by-case analysis, three datasets were chosen: the raw dataset (EN_TEST), the best performing dataset (M3_TEST) and the worst performing dataset (M21_TEST). Figure 8 shows the results on the probability plots as returned for these three datasets in the testing phase of the ANN. The results show how the probability of correctly classifying an observation into a given class differs depending on the median filter used. Figure 8a shows that, without any filtering, the observations characterized by high acceleration values (g) are typically classified as “Drilling”; as such, a high probability was observed for acceleration observations greater than 3 g of being clearly classified as “Drilling“. As the acceleration magnitude decreased, the probability of confusing the “Drilling” with “Other” events begun to grow. The breaking probability point between the two was around 2 g, a level at which the two events could be confused, with probabilities ranging between 40% and 60% for both. This indicates that the results of the classification for this acceleration range of values might be randomly chosen. As the acceleration magnitude continued to decrease, it was obvious that this difference changed in the advantage of the “Other” class, which received a higher chance of occurrence (+90%) in the range of 1.35 to 1.70 g. From 1.35 g below, the “Stopped“ class begun to increase its probability, and for less than 1.22 g, the probability of classifying the observations as “Stopped” has increased significantly.

In Figure 8b, on the other hand, the use of a three-observation window for data filtering has dropped the magnitude of acceleration. The point at which the probabilities vary from 40% to 60% for “Drilling“ and “Other“ events was similar to that found for the EN_TEST dataset, but the ranges of probabilities in which the observations may be confused has decreased. Figure 8c shows the dataset with the worst overall results in terms of classification accuracy and generalization error. It was confirmed that, unlike the other two cases, this one retained a lower probability of categorization for “Drilling” events. On the other hand, “Stopped” class of events has preserved a low probability in the acceleration range higher than 1.41 g and significantly increased its probability in ranges less than 1.41 g, which was a typical result of applying a median filter over a window of 21 observations.

## 4. Discussion

There are practical applications in which machine learning can support the improvement of forest operations in terms of performance. Improvement can be achieved by the use of dedicated tools incorporating specific algorithms that help in decision making. Nevertheless, offline tests on the capability of machine learning techniques might be needed before the real-time implementation of the software and hardware components so as to be able to check which parameters need adjustment and to what extent. The research of [9] demonstrates how to save time, resources and money by implementing offline data analysis systems for a small, manually operated bandsaw. Artificial Neural Networks (ANNs) are just one type of machine learning implementation methodology; acknowledging the wide variety of machine learning techniques, the choice of ANNs for this study was mainly driven by the authors’ experience with them. As previously stated, these algorithms are relying on a collection of interconnected units that have been taught to accept input data, analyze it and provide a result or produce some sort of output values [49]. By the use of machine learning techniques in forestry-related studies, it has been reported that, with the deployment of AI, there is an excellent potential for classification [10,11,13] and regression problems [35].

Some studies [9,10,13] have demonstrated that ANN models can reach a classification accuracy (CA) of close to 100% for some time- and task-based classification applications. Moreover, the classification accuracy (CA) has been found to be one of the most popular metrics used to evaluate the classification performance in several applications [47]. Although several classification performance metrics were computed in this study, only the CA and Log loss metrics were used to prove the hypothesized improvements in terms of performance. To improve the classification performance while keeping the errors at minimum, this work implemented the ANN parameter tuning and signal filtering techniques, an approach that has already been used in forestry applications, although the workflow was different [10,11,13]. In this work, median filtering was done by developing ten new datasets based on the filters’ window size. The testing phase resulted in an improvement of the CA in the M3_TEST and M5_TEST datasets as compared to the EN_TEST data; therefore, the median filtering had a positive effect on the classification accuracy. The regularization parameter tuning, on the other hand, has shown that the best results in terms of generalization errors (Log loss) were those when the regularization parameter was set at α = 0.01. Obviously, this indicates that the choice of the regularization parameter might have a strong influence on the generalization errors. For instance, when α was set at 0.01, the errors were the least for the M3_TEST dataset, which has also shown good results in terms of classification performance during testing. Accordingly, these results stand for the improvements that some sizes of the median filters could bring to the classification performance, which emphasized the highest performance of the M3 filter in terms of both classification performance and generalization error in the testing phase. In turn, the same results could also indicate that other types of operations, which could be monitored by the approach taken in this study, might need different configurations of the ANNs by tunning as well as different median filter sizes, a fact that remains open for research. This is because the use of the datasets filtered by window sizes of three, five and seven observations have generally shown good results in both the training and testing phase, while other median filters taken into study did not perform in the same way. For instance, the M21_TEST dataset has outputted a similar classification accuracy for the “Drilling” event, but it performed poorly when the “Other” class was in question. This behavior may be due to the way that the magnitude of the raw data is rescaled by the filter’s window size. For instance, some events belonging to the class “Stopped” had a long occurrence in the time domain (data not shown herein). For these events, an increased window of the filter (21 observations) has outputted a classification accuracy of 100% by removing most of the noise specific to acceleration data collected in a fixed position (no effects brought by movement or vibration). In contrast, the events from the “Other” class were poorly classified as such (27%), a fact that may be related to the frequent transition from one to other event type as specific to this class. In summary, filter size needs to be carefully tuned to meet the characteristics of the underlying process taken into study.

The location of the devices used to collect the data may be an important factor because the accurate learning of the model may depend on it [8]. Some procedures were proposed for the same type of datalogger to make the learned model invariant to its placement location [13]. In this study, the data came from four days of observations in which the datalogger was placed at roughly the same location on the driller. Obviously, this might have generated a slight variation in data, which was then manipulated by median filtering. It is likely, therefore, that different types of drillers or contrasting operational conditions for the same driller will produce different results. Further studies should address this issue, probably by approaching the problem of data collection and processing as being multimodal, as there are several techniques documented in literature which may support such efforts [50].

Non-ordinary events that occurred during the operations were among the factors that could have been generated confusions in the learning phase of the ANN. The surface of the ground was uneven during the operations, so when the machine was in motion, the driller showed some atypical movements which could have been affected the response in terms of acceleration. These movements, which appeared at random during the work, may have generated stronger acceleration signals. In this case, the model could have learned and interpreted the events that were “Moving” as if they were “Drilling” events. The same is applicable for low-magnitude acceleration signals that emerged during the drilling, since there were moments when the driller was inside the soil but moved slowly, generating lower magnitudes in acceleration, which could have been mistaken as other events.

It is a fact, however, that many ML applications require particular tunning and configurations able to reliably deal with the underlying processes [43] and the characteristics of the collected signals. Nevertheless, the applicability of the procedures used herein may be extended to several other types of forestry-related activities. Already, approaches similar to those described in this study have been tested for motor-manual [8,13] and sawmilling [9] operations. Therefore, with a wise choice of the acceleration sensors’ placement and a careful design of the input signals and of the algorithms used, automatic monitoring could be proven to have a lot of potential in the future of forest operations. For the moment, the applicability of the methods and results given herein is limited to offline applications. However, it has been shown that even offline data handling may be successfully implemented so as to be able to deal with high amounts of data while solving both scientific and practical problems related to the operational monitoring by automated means [9,10,13]. This might not change in the near future, as the contractors of machines and forest equipment are often small companies that are not interested in detailed production monitoring data, being in the search, rather, for cheap solutions to enable their work. Therefore, any purpose-designed data-analytics module added to their machines will only burden them financially. On the other hand, this is also a limitation for the science and for the management of operations, which are lacking the data needed for decision making and improvement. Until reaching a balance so as to have such data readily available and usable, the offline approach could be a viable solution to automated data collection, processing and analysis.

## 5. Conclusions

The main findings of this work demonstrate that using median filters and fine parameter tuning can improve the classification accuracy and decrease the generalization errors when using ANNs to predict the operational performance of pit-drilling operations. However, not all of the window sizes of the median filters led to the same classification performance, with some cases showing poorer results. The highest performance of the median filters with a window size of three observations may be due to the duration of specific events and their frequency distribution on the time scale. Therefore, for other kind of applications which might hold a contrasting distribution of the events on the time scale and in their duration, other window sizes could be more feasible. Nevertheless, evaluating the potential of acceleration signal processing by median filtering in increasing the event-based classification performance is one of the merits of this study, while the approach used herein holds the potential to be transferred to other types of operations.

## Figures and Tables

**Table 1:** Proportion of true classes in the samples.

Date of Collection	Size (s)	Class Size (s)			Class Share (%)		
		Stopped	Drilling	Other	Stopped	Drilling	Other
21/11/18	22,910	2921	11,916	8073	12.7	52.0	35.2
22/11/18	20,870	3651	9031	8188	17.5	43.3	39.2
24/11/18	19,679	6255	8184	5240	31.8	41.6	26.6
27/11/18	20,226	7104	7190	5932	35.1	35.5	29.3
Total	83,685	19,931	36,321	27,433	24.3	43.1	32.6

**Table 2:** Descriptive statistics of misclassified and correctly classified data.

Regularization Term	Dataset	Classes	Total	Correctly Classified		Misclassified	
				N	%	N	%
α = 0.01	EN_TEST	Drilling	9577	9432	98	145	02
		Other	7411	6095	82	1316	18
		Stopped	7104	6243	88	861	12
	M3_TEST	Drilling	9577	9442	99	135	01
		Other	7411	6241	84	1170	16
		Stopped	7104	6539	92	565	08
	M21_TEST	Drilling	9577	9376	98	201	02
		Other	7411	1999	27	5412	73
		Stopped	7104	7096	100	8	0

## Data Availability

The data supporting this study may be available on request to the corresponding author.
